# Instrumented Assessment of Static Balance Parameters and Their Association with Anthropometric Characteristics in Physiotherapy Students

**DOI:** 10.3390/s26206461

**Published:** 2026-10-13

**Authors:** Dragana Vukliš, Rastislava Krasnik, Milena Radulović, Aleksandra Mikov, Jelena Zvekić-Svorcan, Milica Stanić, Dajana Dedić Novaković, Nataša Nenadov Jovanović, Jelena Stupar, Ivana Heinimann

**Affiliations:** 1 Faculty of Medicine, University of Novi Sad, 21000 Novi Sad, Serbia; rastislava.krasnik@mf.uns.ac.rs (R.K.); milena.radulovic@mf.uns.ac.rs (M.R.); aleksandra.mikov@mf.uns.ac.rs (A.M.); jelena.zvekic-svorcan@mf.uns.ac.rs (J.Z.-S.); dajana.dedic-novakovic@mf.uns.ac.rs (D.D.N.); natasa.nenadov-jovanovic@mf.uns.ac.rs (N.N.J.); 903026d25@mf.uns.ac.rs (J.S.); 2 Institute of Child and Youth Health Care of Vojvodina, 21000 Novi Sad, Serbia; 3 Special Hospital for Rheumatic Diseases, 21000 Novi Sad, Serbia; 4 Oncology Institute of Vojvodina, 21204 Sremska Kamenica, Serbia; 5 University Center of Internal Medicine, Department of Internal Medicine, Cantonal Hospital Baselland, 4101 Bruderholz, Switzerland; ivana.heinimann@ksbl.ch

**Keywords:** center of pressure, CoP parameters, Vicon motion capture system, single-leg stance test, static balance, force plate

## Abstract

Balance is a key component of postural stability, and center of pressure (CoP) parameters enable objective assessment of postural control. This study examined associations between anthropometric characteristics and CoP parameters during the Single-Leg Stance Test (SLS) in physiotherapy students. This cross-sectional observational study included 69 students from the Medical Rehabilitation undergraduate degree at the Faculty of Medicine, University of Novi Sad, Serbia, grouped according to year of study (first and fourth). Participants underwent anthropometric measurements and static balance assessment using the SLS with eyes open and closed, with CoP parameters recorded using the Vicon^®^ system and a force plate. CoP excursion in the anteroposterior (AP) and mediolateral (ML) directions and CoP mean velocity (CoP MV) were analyzed using descriptive statistics and Spearman’s correlation (*p* < 0.05). In first-year students, CoP MV was positively correlated with body mass and BMI during right-leg stance with eyes open (*r* = 0.457, *p* < 0.05; *r* = 0.466, *p* < 0.05). In fourth-year students, CoP MV was positively correlated with body height and left lower limb length during left-leg stance with eyes open (*r* = 0.485, *p* < 0.01; *r* = 0.500, *p* < 0.01). Overall, CoP MV demonstrated the greatest number of significant associations. These findings suggest associations between anthropometric characteristics and selected static balance parameters in physiotherapy students.

## 1. Introduction

Balance is one of the basic components of motor function and is crucial for maintaining postural stability, efficient execution of movements, and safe performance of daily activities. Static balance refers to the ability to maintain a stable body stance in conditions without movement, while dynamic balance implies the ability to maintain postural control during motion and adapt to changes in external or internal forces [1,2]. Maintenance of static balance is based on the integration of visual, vestibular, and somatosensory information to ensure that the center of mass (CoM) remains within the base of support (BoS) [2]. Objective assessment of postural control typically involves the analysis of center of pressure (CoP) parameters, which represent sensitive indicators of neuromuscular function and enable the identification of subtle changes in stability that cannot be observed through clinical examination [2,3,4]. The modern gold standard for the objective assessment of static balance and postural control relies on force plate-based measurements of center of pressure (CoP), while motion analysis systems with infrared cameras can provide complementary information on body movement and postural control [1,5]. Force plates remain the reference technology for quantifying postural stability, with CoP being one of the most widely used objective measures of postural control [6]. The Single-Leg Stance Test (SLS) plays a particularly prominent role in this process, as it assesses the ability to maintain balance on each lower limb separately, with the possibility of detecting postural asymmetries [2,3,4]. Parameters such as CoP excursion in the anteroposterior (AP) and mediolateral (ML) directions, as well as CoP mean velocity (CoP MV) are reliable indicators of the quality of postural control and stability [1,2,5]. The static balance attributes are influenced by numerous individual factors, among which anthropometric characteristics play a significant biomechanical role. Body mass, body height, body mass index (BMI), lower limb length, transverse diameter of the knee and ankle joints can change the CoM position, the magnitude of force moments, and balance maintenance strategies [7]. Although the relationships between anthropometric characteristics and postural stability have been examined in different populations, prior studies have yielded inconsistent results, and in particular there is a lack of data on the association of these characteristics with objective CoP parameters in young healthy individuals [5,7,8,9,10]. Recent evidence further supports the relevance of anthropometric characteristics for the interpretation of CoP measures. Paterna et al. [11] demonstrated that body weight and height were significantly associated with the magnitude of CoP displacement during postural perturbations in healthy adults, whereas the latency and duration of the CoP response were not significantly influenced by anthropometric characteristics or sex. These findings suggest that body size may contribute to variability in CoP-based measures and should therefore be considered when interpreting objective postural control parameters. However, the cited study examined responses to external trunk perturbations, whereas evidence regarding the relationship between anthropometric characteristics and CoP parameters during static single-leg stance remains limited.

Physiotherapy students represent a specific population of future healthcare workers whose profession requires a high level of postural control, neuromuscular coordination, and physical ability [10,12]. During their education, they acquire knowledge of anatomy, biomechanics, posture assessment, and kinesitherapy, but also spend long periods in a seated position as well as participate in practical teaching and manual techniques, exposing them to various static loads that can affect the development of postural patterns. Since physiotherapists are among the professional groups with an increased risk of developing work-related musculoskeletal disorders (WMSDs), early recognition of changes in postural control and the factors that influence it can be the basis for risk assessment and the implementation of preventive measures already during studies [13]. Objective assessment of balance can contribute to the identification of students with an increased risk for the development of functional disorders in the future and represents the foundation for planning preventive and educational interventions [10,12,13].

Previous research has examined the relationships between anthropometric characteristics and postural stability in different populations, but findings regarding these associations have been inconsistent, particularly when objective CoP parameters are considered [10,12,13]. In physiotherapy students, the relationship between anthropometric characteristics and specific CoP parameters during single-leg stance remains insufficiently investigated [3,5,7]. In particular, further evidence is needed regarding the associations of body mass, body height, BMI, lower limb length, and knee and ankle joint dimensions with AP and ML CoP excursion and CoP MV under eyes-open and eyes-closed conditions. Therefore, examining these relationships in physiotherapy students, including students at different stages of their education, may provide additional information on the interaction between anthropometric characteristics and instrumented measures of static balance.

Therefore, the aim of this study was to examine the association of anthropometric characteristics (body mass, body height, BMI, lower limb length, transverse knee and ankle joint diameter) with CoP parameters (AP and ML CoP excursion, CoP MV) during the SLS with eyes open and closed, evaluated using the motion analysis system and force plate. We hypothesized that anthropometric characteristics would be significantly associated with selected CoP parameters during static single-leg stance in physiotherapy students.

## 2. Materials and Methods

This cross-sectional observational study included 69 students enrolled in the Medical Rehabilitation study program at the Faculty of Medicine, University of Novi Sad, Serbia, who were divided into two groups based on the study year (Year 1 and Year 4). The research was conducted from October to December 2025. All students enrolled in the first and fourth years of the Medical Rehabilitation study program were invited to participate in the study. Participation was voluntary, and students were included if they met the predefined eligibility criteria and provided informed consent. Students who did not meet the eligibility criteria or declined participation were not included in the final sample. The exact number of students who declined participation was not systematically recorded and therefore cannot be reliably reported.

The minimum required sample size was calculated prior to data collection using the proportion estimation formula. Based on an 80% confidence level, an expected proportion of 0.50, and a maximum allowable margin of error of 0.08, at least 64 participants were required. Given that the primary analytical objective included examining associations between anthropometric characteristics and static balance parameters, an additional approximate sensitivity analysis for Spearman’s rank correlation was performed. At a two-sided significance level of α = 0.05 and 80% statistical power, the total sample of 69 participants corresponded approximately to a detectable correlation of |ρ| = 0.34. For subgroup analyses according to year of study, the sample sizes of 25 and 44 participants corresponded approximately to detectable correlations of |ρ| = 0.55 and |ρ| = 0.42, respectively.

All potential participants were familiarized with the research procedures before inclusion. After signing the informed consent form for participation in the study and the consent form for photography and video recording during testing, students completed a questionnaire specifically developed for this study. Subsequently, each participant’s static balance was assessed under laboratory conditions. The study was conducted in accordance with the principles of the Declaration of Helsinki, and the research protocol was approved by the Ethics Committee of the Faculty of Medicine, University of Novi Sad, Republic of Serbia (Approval No. 01-39/245/1, 25 June 2024).

The inclusion criteria were: enrolment in the Medical Rehabilitation study program, age 18–30 years, and voluntary informed consent to participate in the research. Criteria for exclusion from the study included: enrolment in other study programs; termination of student status; surgical treatment of the lower-limb joints (hip, knee, and/or ankle) in the last six months; presence of acute or chronic lower limb injuries that may affect postural control; presence of neuromuscular diseases, vestibular disorders or other medical conditions that may affect the ability to maintain balance; the inability to properly perform the testing protocol.

Participants’ static balance was assessed at the Laboratory for Mechatronics and Robotics, Faculty of Technical Sciences, University of Novi Sad, Serbia, where the SLS was performed using the Vicon^®^ motion analysis system (2020, Vicon Motion Systems© Ltd., Oxford, UK, registered no. 1801446). Testing with the motion analysis system involved securing a total of 18 reflective markers (14 mm in diameter) to the participants’ lower extremities using self-adhesive tape and recording their movements using eight T160 infrared cameras operating at 200 Hz. The marker configuration consisted of the standard Plug-in Gait lower-body markers (Plug-in Gait Module^®^, version 3.3.1, Vicon Motion Systems© Ltd., Oxford, UK): left and right anterior superior iliac spine (LASI, RASI), left and right posterior superior iliac spine (LPSI, RPSI), left and right thigh (LTHI, RTHI), left and right lateral knee (LKNE, RKNE), left and right tibia (LTIB, RTIB), left and right ankle (LANK, RANK), left and right heel (LHEE, RHEE), and left and right toe (LTOE, RTOE). In addition, one marker was placed on the dorsal aspect of each great toe for research purposes. Marker placement was performed according to the manufacturer’s Plug-in Gait marker placement guidelines [14]. Marker trajectories were recorded and reconstructed in three-dimensional space using the motion analysis system.

To assess postural stability, an AMTI Optima BMS400600 multi-component force plate (Advanced Mechanical Technology, Inc., Watertown, MA, USA) synchronized with the Vicon^®^ motion analysis system was used. The force plate had a top surface of 600 × 400 mm^2^ and a vertical profile thickness of 41.4 mm. Force-platform data were acquired at a sampling frequency of 1000 Hz. For analog force-platform data, a low-pass digital Butterworth filter was applied using a fourth-order, zero-lag filter with a cut-off frequency of 300 Hz. According to the Vicon Nexus (version 2.14; Vicon Motion Systems© Ltd., Oxford, UK) processing procedure, this filtering applies to the calculated Force and Moment signals, whereas Raw and Center of Pressure (CoP) signals cannot be filtered using this function. Therefore, no additional filtering was applied to the CoP signals prior to calculation of CoP mean velocity (MV). CoP MV and total anteroposterior (AP) and mediolateral (ML) CoP excursion were calculated from the recorded CoP data. All measurements were carried out according to a predefined protocol, under the same laboratory conditions, and by the same research team.

The questionnaire designed for the purposes of this research aimed to capture the following data: age, sex, place of residence, year of study, body mass (kg), body height (cm), and dominant leg. Leg dominance was determined based on participants’ self-reported preferred leg for kicking a ball. BMI was calculated as the ratio of body mass (kg) to the square of body height (m^2^). The body mass was classified according to the World Health Organization (WHO) criteria (available at: https://www.who.int/data/gho/data/themes/topics/topic-details/GHO/body-mass-index?introPage=intro_3.html (accessed on 3 August 2026)).

Before testing, the total lower limb length, as well as the transverse knee and ankle joint diameter (cm) were measured for each participant. For the total lower limb length measurement, the participants were instructed to lie on a bed in the supine position, and the distance from the anterior superior iliac spine (ASIS) to the medial malleolus was recorded using a centimeter tape. The transverse knee and ankle joint diameter was measured using a metal caliper (Small Metal Anthropometer–Bone Caliper, model 01291A, Lafayette Instrument Company, Lafayette, IN, USA), in accordance with the manufacturer’s instructions (available at: https://www.youtube.com/watch?v=fl7CWDmIaHw (accessed on 5 August 2026)). During measurements, the caliper was placed on the bony prominences of the knee joint line (on the lateral and medial sides), as well as on the bony prominences in the area of the ankle joint (on the lateral and medial malleolus), and the values are expressed in cm.

### 2.1. Static Balance Assessment

**Figure 1:**
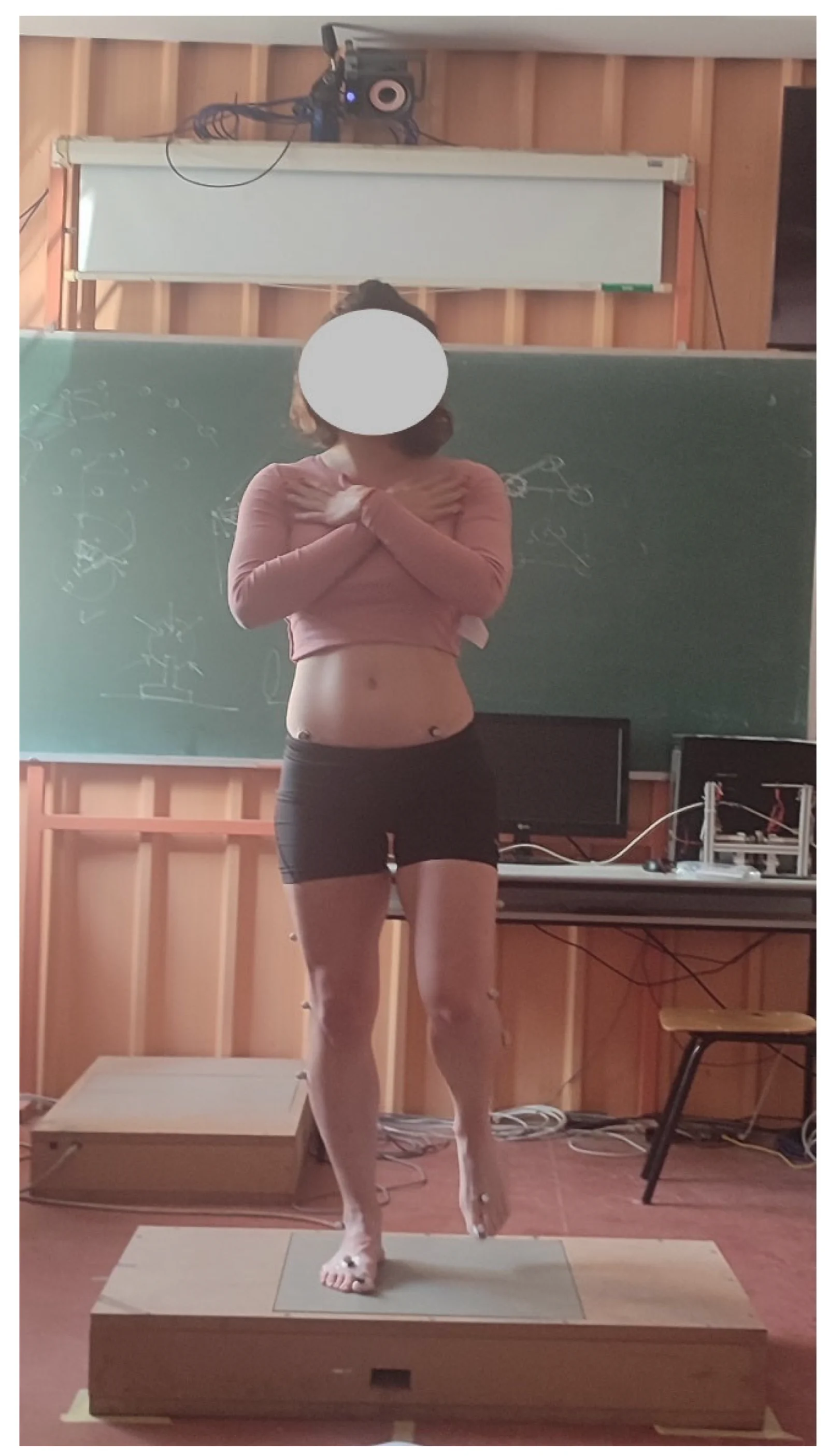
Static balance assessment using a force plate and Vicon^®^ motion analysis system (original photograph by the author).

The Single-Leg Stance Test (SLS) was conducted to assess static balance with eyes open and closed under laboratory conditions. Prior to testing, participants were given one trial attempt to familiarize themselves with the test procedure. The testing order was standardized and identical for all participants: right-leg stance with eyes open, left-leg stance with eyes open, right-leg stance with eyes closed, and left-leg stance with eyes closed. A one-minute break was provided between consecutive trials. During the test, participants stood barefoot on one leg on the force plate while the contralateral leg was raised, ensuring that the foot did not touch the ground. The arms were crossed on the chest (Figure 1). Before each measurement, participants received standardized verbal instructions on how to perform the test, and the entire procedure was demonstrated by the examiner. Using a digital stopwatch (Flott^®^ F-396), stance duration was measured in seconds, starting when the participant lifted the non-supporting foot from the floor following the verbal command to begin the trial and ending when the raised foot touched the ground, the foot of the supporting leg or an arm moved, the eyes were opened during the eyes-closed condition, or a maximum time of 60 s had elapsed [3,4]. The stance duration was determined during testing using the digital stopwatch and was not derived from the Vicon motion-capture data. The duration of each SLS trial was recorded individually using the digital stopwatch and corresponded to the actual time elapsed from trial initiation until the predefined termination criterion was reached or the maximum duration of 60 s was completed. The test is freely available at: https://www.physio-pedia.com/Single_Leg_Stance_Test (accessed on 6 August 2026).

The data recorded during the SLS were used for analysis. Data obtained during testing were captured using a Vicon^®^ motion analysis system and a force plate and exported to Microsoft Excel 2016 (Microsoft Corporation, Redmond, WA, USA) for further processing. The recorded data contained CoP coordinates in the X, Y, and Z axes. The X and Y coordinates, representing the horizontal plane of the force plate, were used to calculate the CoP parameters, whereas the Z coordinate was not included in the calculations. For each participant, the entire recording interval corresponding to the individual SLS trial was analyzed, from the start of the test until the predefined termination criterion was reached or until the maximum duration of 60 s was completed. Thus, the analyzed time window was based on the actual duration of each individual SLS trial rather than on a fixed duration across participants. The duration of the analyzed interval corresponded to the SLS stance duration measured with the digital stopwatch. For CoP mean velocity, the total CoP path length was divided by the actual duration of the analyzed recording, thereby accounting for differences in trial duration. CoP excursion parameters were calculated over the same individual recording interval. The following CoP parameters were calculated from the recorded X and Y coordinates.

CoP excursion in the anteroposterior (AP) direction was calculated as the difference between the maximum and minimum CoP values along the *Y*-axis:

[CoP\excursion_{AP} = max(Y) − min(Y)],

CoP excursion in the mediolateral (ML) direction was calculated as the difference between the maximum and minimum CoP values along the *X*-axis:

[CoP\excursion_{ML} = max(X) − min(X)],

To calculate CoP mean velocity (CoP MV), the total CoP path length was first calculated as the cumulative Euclidean distance between consecutive CoP positions:

[Path\Length = \sum_{i = 1}^{n − 1}\sqrt{(X_{i + 1} − X_i)^2 + (Y_{i + 1} − Y_i)^2}],

CoP mean velocity was subsequently calculated as:

[CoP\Mean\Velocity = \frac{Path\Length}{Total\Time}],

where *Total Time* represents the duration of the analyzed recording in seconds. CoP excursion was expressed in millimeters (mm), and CoP mean velocity in millimeters per second (mm/s). AP and ML CoP excursion and CoP MV were calculated separately for the right (R) and left (L) legs under eyes-open (EO) and eyes-closed (EC) conditions.

For the SLS test, the outcome measure was the duration of single-leg stance, expressed in seconds (s), recorded separately for the right and left legs under EO and EC conditions. Absolute difference (diff) values were also calculated and expressed in seconds (s): the difference between the right and left legs under EO conditions, the difference between the right and left legs under EC conditions, the difference between EO and EC conditions for the right leg, and the difference between EO and EC conditions for the left leg.

### 2.2. Statistical Analysis

Information captured during tests was analyzed using descriptive and inferential statistics. The normality of data distributions was evaluated through the Shapiro–Wilk and Kolmogorov–Smirnov normality tests, which indicated that most variables deviated from a normal distribution. Accordingly, continuous variables were presented as median (Me), interquartile range (IQR), minimum (Min), and maximum (Max) values. The relationship between static balance parameters and anthropometric characteristics was examined using Spearman’s correlation coefficient. The level of statistical significance was set at *p* < 0.05. All analyses were performed in IBM SPSS Statistics for Windows, Version 29.0 (IBM Corp., Armonk, NY, USA).

## 3. Results

The research sample consisted of 69 students, aged 19 to 25 years (median = 23 years, IQR = 1.0), 82.6% of whom were female and 17.4% were male, whereby 36.2% and 63.8% were in the first and the fourth year of study, respectively. At 95.7% of the sample, those living in urban areas predominated, with the remaining 4.3% residing in rural areas. The median body mass of the participants was 62.0 kg (IQR = 16.0), with values ranging from 42.0 to 115.0 kg. The median body height was 170.0 cm (IQR = 8.0), spanning from 157.0 to 202.0 cm. The median BMI was 21.7 kg/m^2^ (IQR = 3.2), while the recorded values ranged from 15.8 to 31.9 kg/m^2^. According to the WHO classification, the largest number of participants had a normal body mass (73.9%), 17.4% were overweight, while underweight and obesity were noted in 4.3% of the cases each. The median total length of both right and left leg was measured at 90.0 cm (IQR = 5.0). The median transverse diameter of the right and the left knee was 10.0 cm (IQR = 0.9) and 10.0 cm (IQR = 0.8), respectively. The median transverse right and left ankle joint diameter was 6.4 cm (IQR = 0.4) and 6.5 cm (IQR = 0.4), respectively. The right leg was dominant in 88.4% of participants (Table 1).

The duration of the SLS trials varied according to the predefined termination criteria. Trial duration ranged from 15 to 60 s for the right-leg eyes-open condition, from 3 to 60 s for the left-leg eyes-open condition, and from 1 to 60 s for both the right- and left-leg eyes-closed conditions.

The relationship between anthropometric characteristics and static balance parameters in the first-year students was evaluated through Spearman’s correlation analysis. Significant associations were observed across several static balance parameters. The findings involving CoP mean velocity (CoP-MV) represented the most consistent pattern of associations, while additional individual associations were observed for SLS performance and CoP excursion in the mediolateral direction, as detailed below. The results showed that the difference in the SLS results between the right and left leg with eyes open (SLS-diff-R/L-EO) was in a statistically significant positive correlation with the transverse right knee joint diameter (*r* = 0.436; *p* < 0.05) as well as the transverse left knee joint diameter (*r* = 0.445; *p* < 0.05). CoP excursion in the ML direction when tested on the right leg with eyes open was in a statistically significant negative correlation with the transverse right ankle joint diameter (*r* = −0.519; *p* < 0.01), while when tested on the right leg with eyes closed it was in a statistically significant positive correlation with the transverse right knee joint diameter (*r* = 0.409; *p* < 0.05). CoP excursion in the ML direction when tested on the left leg with eyes closed was in a statistically significant negative correlation with BMI (*r* = −0.456; *p* < 0.05) and the transverse left knee joint diameter (*r* = −0.535; *p* < 0.01).

CoP MV when tested on the right leg with eyes open was in a statistically significant positive correlation with body mass (*r* = 0.457; *p* < 0.05), BMI (*r* = 0.466; *p* < 0.05), as well as the transverse right knee joint (*r* = 0.453; *p* < 0.05) and left knee joint (*r* = 0.516; *p* < 0.01) diameter. CoP MV when tested on the left leg with eyes open had a statistically significant positive correlation with the transverse left knee joint diameter (*r* = 0.429; *p* < 0.05). When tested on the right leg with eyes closed, CoP MV was in a statistically significant positive correlation with body mass (*r* = 0.400; *p* < 0.05) and BMI (*r* = 0.397; *p* < 0.05). Likewise, when tested on the left leg with eyes closed, CoP MV was in a statistically significant positive correlation with body mass (*r* = 0.511; *p* < 0.01) and BMI (*r* = 0.553; *p* < 0.01). No statistically significant correlations (*p* > 0.05) were found for the remaining parameters (Table 2).

The relationship between anthropometric characteristics and static balance parameters in the fourth-year students was also examined by conducting Spearman’s correlation analysis. Statistically significant associations were determined for the CoP MV parameter only, representing the predominant pattern of findings in this group. The individual significant associations with specific anthropometric characteristics and testing conditions are presented below. When tested on the right leg with eyes open, CoP MV was in a statistically significant positive correlation with body mass (*r* = 0.430; *p* < 0.01), body height (*r* = 0.412; *p* < 0.01), BMI (*r* = 0.403; *p* < 0.01), right lower limb length (*r* = 0.356; *p* < 0.05), left lower limb length (*r* = 0.392; *p* < 0.01), transverse right ankle joint diameter (*r* = 0.401; *p* < 0.01), and transverse left ankle joint diameter (*r* = 0.336; *p* < 0.05). CoP MV when tested on the left leg with eyes open was in a statistically significant positive correlation with body mass (*r* = 0.426; *p* < 0.01), body height (*r* = 0.485; *p* < 0.01), BMI (*r* = 0.350; *p* < 0.05), right lower limb length (*r* = 0.451; *p* < 0.01), left lower limb length (*r* = 0.500; *p* < 0.01), and transverse right ankle joint diameter (*r* = 0.378; *p* < 0.05).

CoP MV when tested on the right leg with eyes closed was in a statistically significant positive correlation with body mass (*r* = 0.323; *p* < 0.05), BMI (*r* = 0.325; *p* < 0.05), as well as the transverse right ankle joint (*r* = 0.404; *p* < 0.01) and left ankle joint (*r* = 0.444; *p* < 0.01) diameter. When tested on the left leg with eyes closed, it was in a statistically significant positive correlation with body mass (*r* = 0.356; *p* < 0.05), body height (*r* = 0.425; *p* < 0.01), BMI (*r* = 0.297; *p* < 0.05), right lower limb length (*r* = 0.307; *p* < 0.05), left lower limb length (*r* = 0.317; *p* < 0.05), and the transverse right ankle joint (*r* = 0.420; *p* < 0.01) and left ankle joint (*r* = 0.405; *p* < 0.01) diameter. No statistically significant correlations (*p* > 0.05) were found for the other analyzed parameters (Table 3).

## 4. Discussion

In this research, the relationship between the anthropometric characteristics and the static balance parameters pertaining to the cohort of first- and fourth-year physiotherapy students was analyzed using the Single-Leg Stance Test (SLS) results as well as the objective center of pressure (CoP) parameters. Assessing postural control in future physiotherapists is of particular importance, as their professional activity upon graduation includes exposure to prolonged static loads, manual techniques, and various working positions that require precise body control [10,13,15,16].

Our research sample consisted of 69 students, with an average age of 23 years, 82.6% of whom were female. A similar sample composition was described by Tivadar et al., where the female gender also dominated among physiotherapy students, as is the case for most study programs in health sciences [17]. The anthropometric characteristics of our research participants corresponded to the population of young healthy adults, which is in accordance with the findings of prior studies conducted among students enrolled in such study programs [17,18].

The analyses performed as a part of the present study revealed a link between certain anthropometric characteristics and static balance parameters, whereby the connection patterns differed between the first- and the fourth-year students. In the first-year cohort, anthropometric characteristics were associated with a greater number of CoP excursion indicators, especially in the mediolateral direction (ML), while in their fourth-year counterparts, significant associations were predominantly established for the CoP mean velocity (CoP MV). These findings indicate that body composition may represent one of the factors affecting postural control, but its influence depends on the type of analyzed CoP parameter and the task demands [19,20,21,22,23].

Balance during one-legged stance is a result of the integration of the sensory system, proprioception, vestibular information, and neuromuscular control [24,25,26]. Kapan et al. state that balance depends on different postural control strategies that are activated according to the demands of the task being performed [18]. Accordingly, the fact that in our study not all CoP parameters were associated with anthropometric characteristics can be explained by the different control mechanisms involved in AP and ML stability. Similar findings were reported by Blaszczyk et al., who showed that certain parameters of postural sway during the SLS were associated with body mass, body height, BMI, and foot dimensions, whereby the association was not equal for all stability parameters [19]. Rizzato et al. [8,9], Gonçalves et al. [20], and Redfern et al. [27] further indicate that CoP MV represents a significant and sensitive indicator of postural control, as it reflects the speed of corrective adjustments and the interaction among biomechanical characteristics, sensorimotor integration, and neuromuscular control. In our research, this parameter exhibited the greatest number of statistically significant associations with anthropometric characteristics, particularly body mass, BMI, body height, and lower extremity attributes.

The influence of anthropometric characteristics on postural control can be explained by biomechanical changes that occur due to different body proportions. These findings are also in line with the recent study by Paterna et al. [11], which demonstrated that body weight and height were associated with the magnitude of CoP displacement during externally induced postural perturbations in healthy adults. Although their protocol differed from the static single-leg stance used in the present study, both studies support the relevance of anthropometric characteristics when interpreting CoP-based measures of postural control. Greater body mass, longer limbs, or larger dimensions of certain segments can affect the CoP position and require greater corrective reactions to maintain stability [8,9,24,25]. According to Rizzato et al., CoP MV represents a significant indicator of the postural adjustment efficacy, as it reflects the interaction between biomechanical characteristics and neuromuscular control [8]. Rizzato et al. ascribed similar importance to the CoP parameters, indicating that more demanding balance tasks can lead to the greater engagement of control mechanisms [9].

Our results are consistent with the findings reported by Alonso et al., who showed that body height and body proportions can influence postural sway parameters in young adults [23]. However, unlike Ferreira et al., who noted a limited influence of body mass and BMI in individuals with normal BMI values [21], in our study these characteristics were associated with certain CoP parameters. This discrepancy may be due to different assessment protocols, as the SLS is a more demanding unilateral task with a reduced base of support (BoS) compared to the dynamic tests used in their research. The influence of anthropometric characteristics on unilateral balance control is also confirmed by Neji et al., who emphasized the importance of body proportions during the Y Balance Test [22]. Although dynamic balance was in focus of their research, while the tests conducted in the present study assessed static balance, the common factor is the need for precise control of body mass (CoM) above reduced BoS. In addition, Abdelmohsen et al. underscored the importance of strategies involving ankle and hip adjustments in maintaining postural balance, which may explain why certain anthropometric characteristics affect only some of the CoP parameters [24].

Differences between the first- and the fourth-year students who took part in our investigation may reflect differences in postural control strategies between the two academic-year groups. Although theoretical education, practical teaching, and clinical experience may potentially contribute to the development of postural control, the cross-sectional design of the present study does not allow these differences to be attributed to educational exposure. Other factors, such as age, physical activity, previous sports participation, sex distribution, and other unmeasured characteristics, may also contribute to the observed differences. Therefore, the findings should be interpreted as differences between academic-year groups rather than as evidence of changes resulting from physiotherapy education [10,12,28]. Glista et al. reported that education and practical activities may be associated with changes in postural characteristics and ergonomic patterns in future physiotherapists [10]; however, longitudinal studies are needed to determine whether similar changes occur over the course of physiotherapy education.

Objective CoP parameter assessment may have potential value for identifying individual characteristics associated with postural stability and could be investigated as a component of preventive approaches during physiotherapy education. However, the present study did not evaluate the clinical utility or effectiveness of instrumented balance assessment for screening, monitoring, or planning preventive interventions. Therefore, these potential applications should be considered as hypotheses for future research. In addition to objective stabilometric assessment, previous research in this domain indicates that combining functional and objective balance tests may provide a more comprehensive insight into postural control in physiotherapy students [29]. During work, physiotherapists conduct a wide range of manual techniques and are often required to maintain the same body positions for a long time while experiencing physical loads [10,13,15,16].

The SLS is a valid approach for assessing static postural control, as it necessitates the integration of somatosensory, visual, and vestibular information. Lin et al. demonstrated that the ability to maintain balance during the SLS can be objectively assessed by examining the quantitative postural oscillation parameters [5]. Accordingly, our results suggest that different CoP parameters may provide complementary information on postural control, with spatial parameters describing the direction and magnitude of postural oscillations, while CoP MV reflects the velocity of CoP displacement.

Although foot structure and proprioceptive abilities were not the subject of our research, their potential role should be considered in future studies, bearing in mind that the foot represents an important source of sensory input for balance control [25,26]. Authors of future studies should adopt a longitudinal design and include additional biomechanical indicators in their analyses to better understand factors contributing to differences in postural stability in physiotherapy students.

This study has several limitations that should be considered when interpreting the reported results. The relatively small number of participants limits the generalizability of our findings, and the relatively small subgroup sizes should also be considered when interpreting the results for first- and fourth-year students. The predominantly female composition of the sample (82.6%) should also be considered when interpreting the findings. Previous research has indicated that sex may be associated with differences in postural control and center-of-pressure characteristics. Paterna et al. reported that anthropometry, age, and sex may influence center-of-pressure displacement during balance testing [11], while Trajković et al. demonstrated sex-related differences in postural stability during single-leg quiet stance in highly trained athletes [15]. In addition, sex-related differences in trunk muscle morphological, functional, and contractile characteristics have been reported in young athletes, suggesting that neuromuscular characteristics may represent another biological factor potentially relevant to postural control [30]. However, sex was not included as a covariate in the present analysis, and sex-related neuromuscular characteristics were not assessed. Therefore, the extent to which the observed associations may differ according to sex cannot be determined from the present data. The unequal sex distribution may also limit the generalizability of the findings to physiotherapy student populations with a more balanced sex distribution. Moreover, certain factors that can significantly affect static balance–such as hip, knee, and foot deformities; foot type and position during testing; position of the lower extremities; and presence of swelling–were not evaluated. Likewise, muscle strength parameters (which necessitate manual muscle testing) or range of joint motion were not assessed as a part of this study. In addition, the characteristics of the support surface on which the SLS was performed were not specifically analyzed, although they may affect the measurement results. Due to the cross-sectional study design, it was not possible to track changes in balance over time. An additional limitation stems from the fact that only one test was used for static balance assessment, while dynamic balance parameters were not included in the analysis. Furthermore, the SLS conditions were performed in a fixed, non-randomized order for all participants (right leg–eyes open, left leg–eyes open, right leg–eyes closed, and left leg–eyes closed). Therefore, potential order, practice, and fatigue effects cannot be completely excluded. Although a familiarization trial and a standardized one-minute rest interval between consecutive trials were provided, the fixed testing sequence may have influenced the performance in subsequent conditions. In addition, only one recorded trial was used for each SLS condition. Therefore, within-condition variability and test–retest reliability could not be assessed. Furthermore, the large number of statistical comparisons increases the possibility that some statistically significant associations may have occurred by chance; therefore, the individual associations should be interpreted cautiously. These shortcomings can be addressed through future research focusing on a larger and more representative sample of subjects, a more detailed assessment of all factors that may affect balance, and a longitudinal follow-up to better understand changes in static balance over time and determine their clinical relevance.

## 5. Conclusions

Based on the findings of this study, statistically significant associations were identified between individual anthropometric characteristics and selected static balance parameters in physiotherapy students. The most pronounced associations were observed for CoP mean velocity, which in both study groups was positively associated with body mass and body mass index, while in fourth-year students, additional associations were observed with body height, lower extremity length, and transverse ankle joint diameter. CoP excursion in the mediolateral direction and SLS performance showed fewer significant associations with anthropometric characteristics. Overall, CoP mean velocity demonstrated the greatest number of significant associations among the assessed static balance parameters. These findings suggest that anthropometric characteristics should be considered when interpreting objective measures of static postural control in physiotherapy students. Further research is warranted to determine whether instrumented balance assessment may have potential value for monitoring postural control and informing preventive approaches during professional education.

## Figures and Tables

**Table 1:** Sociodemographic and anthropometric characteristics of the study participants.

	N = 69
Sex (%)	
Female	57 (82.6)
Male	12 (17.4)
Place of residence (%)	
Urban	66 (95.7)
Rural	3 (4.3)
Year of study (%)	
First year	25 (36.2)
Fourth year	44 (63.8)
Age (years), Me (IQR) Min–Max	23.0 (1.0) 19.0–25.0
Body mass (kg), Me (IQR) Min–Max	62.0 (16.0) 42.0–115.0
Body height (cm), Me (IQR) Min–Max	170.0 (8.0) 157.0–202.0
BMI (kg/m^2^), Me (IQR) Min–Max	21.7 (3.2) 15.8–31.9
BMI categories, n (%)	
Underweight (<18.5 kg/m^2^)	3 (4.3)
Normal weight (18.5–24.9 kg/m^2^)	51 (73.9)
Overweight (25.0–29.9 kg/m^2^)	12 (17.4)
Obesity (≥30.0 kg/m^2^)	3 (4.3)
Dominant leg, n (%)	
Right	61 (88.4)
Left	8 (11.6)
Lower limb length (cm), Me (IQR) Min–Max	
Right	90.0 (5.0) 80.0–106.0
Left	90.0 (5.0) 81.0–106.0
Transverse knee joint diameter (cm), Me (IQR) Min–Max	
Right	10.0 (0.9) 7.0–15.0
Left	10.0 (0.8) 8.3–14.9
Transverse ankle joint diameter (cm), Me (IQR) Min–Max	
Right	6.4 (0.4) 5.8–7.4
Left	6.5 (0.4) 5.8–8.3

Note: N—number of participants; Me—median; IQR—interquartile range; Min—minimum; Max—maximum; BMI—body mass index.

**Table 2:** Correlation between anthropometric characteristics and static balance parameters in first-year students.

	Body Mass (kg)	Body Height (cm)	BMI (kg/m^2^)	Lower Limb Length Right (cm)	Lower Limb Length Left (cm)	TKJD-R (cm)	TKJD-L (cm)	TAJD-R (cm)	TAJD-L (cm)
**SLS-R-EO (s)**	−0.079	−0.087	−0.085	−0.132	−0.084	−0.329	−0.342	−0.059	0.030
**SLS-L-EO (s)**	−0.142	0.241	−0.227	0.071	0.156	−0.255	−0.256	0.286	0.043
**SLS-diff-R/L-EO (s)**	0.147	−0.054	0.195	0.082	−0.007	0.436 *	0.445 *	−0.110	−0.051
**SLS-R-EC (s)**	−0.238	−0.010	−0.316	−0.054	0.077	−0.373	−0.342	−0.076	−0.001
**SLS-L-EC (s)**	−0.118	−0.062	−0.128	0.007	0.079	−0.085	−0.045	−0.135	−0.028
**SLS-diff-R/L-EC (s)**	−0.135	0.041	−0.177	−0.110	−0.067	−0.344	−0.346	0.102	0.090
**SLS-diff-EO/EC-R (s)**	0.129	−0.104	0.209	−0.080	−0.205	0.181	0.150	−0.024	−0.107
**SLS-diff-EO/EC-L (s)**	0.058	0.141	0.039	0.013	−0.024	−0.010	−0.052	0.240	0.035
**CoP-AP-R-EO (mm)**	−0.085	−0.021	−0.042	0.049	−0.025	0.180	0.176	−0.228	−0.168
**CoP-AP-L-EO (mm)**	0.137	−0.072	0.205	−0.173	−0.224	0.115	0.270	−0.250	−0.144
**CoP-AP-R-EC (mm)**	−0.048	0.008	−0.006	0.066	−0.052	0.235	0.110	−0.050	−0.193
**CoP-AP-L-EC (mm)**	−0.142	0.065	−0.169	−0.012	−0.047	−0.230	−0.372	0.063	−0.086
**CoP-ML-R-EO (mm)**	−0.191	−0.215	−0.065	−0.198	−0.245	0.219	0.125	−0.519 **	−0.318
**CoP-ML-L-EO (mm)**	−0.115	−0.117	−0.126	−0.042	−0.125	−0.061	−0.016	−0.363	−0.266
**CoP-ML-R-EC (mm)**	0.244	0.174	0.276	0.184	0.089	0.409 *	0.382	0.163	0.034
**CoP-ML-L-EC (mm)**	−0.378	0.004	−0.456 *	0.087	0.119	−0.372	−0.535 **	0.081	−0.115
**CoP-MV-R-EO (mm/s)**	0.457 *	0.239	0.466 *	0.104	0.047	0.453 *	0.516 **	0.323	0.235
**CoP-MV-L-EO (mm/s)**	0.394	0.189	0.388	0.145	0.136	0.384	0.429 *	0.227	0.268
**CoP-MV-R-EC (mm/s)**	0.400 *	0.238	0.397 *	0.056	0.001	0.213	0.283	0.296	0.132
**CoP-MV-L-EC (mm/s)**	0.511 **	0.142	0.553 **	0.031	−0.007	0.343	0.353	0.374	0.299

Note: Values are presented as Spearman’s correlation coefficients (ρ). TKJD-R—transverse knee joint diameter right; TKJD-L—transverse knee joint diameter left; TAJD-R—transverse ankle joint diameter right; TAJD-L—transverse ankle joint diameter left; SLS—Single-Leg Stance Test; R—right; L—left; EO—eyes open; EC—eyes closed; diff—difference; R/L—right/left; EO/EC—eyes open/eyes closed; s –seconds; mm—millimeters; mm/s—millimeters per second; CoP—center of pressure; AP—anteroposterior; ML—mediolateral; MV—mean velocity; BMI—body mass index; * *p* < 0.05; ** *p* < 0.01.

**Table 3:** Correlation between anthropometric characteristics and static balance parameters in fourth-year students.

	Body Mass (kg)	Body Height (cm)	BMI (kg/m^2^)	Lower Limb Length Right (cm)	Lower Limb Length Left (cm)	TKJD-R (cm)	TKJD-L (cm)	TAJD-R (cm)	TAJD-L (cm)
**SLS-R-EO (s)**	−0.258	−0.161	−0.252	−0.206	−0.193	−0.146	−0.203	−0.091	−0.085
**SLS-L-EO (s)**	0.076	0.006	0.071	−0.134	−0.151	0.084	0.068	0.092	0.066
**SLS-diff-R/L-EO (s)**	0.073	0.133	0.057	0.193	0.195	0.045	0.080	0.088	0.152
**SLS-R-EC (s)**	−0.023	0.116	−0.094	0.085	0.119	0.059	0.019	−0.114	−0.114
**SLS-L-EC (s)**	−0.058	−0.069	−0.038	−0.165	−0.108	0.042	0.045	0.022	0.067
**SLS-diff-R/L-EC (s)**	−0.002	0.044	0.001	−0.083	−0.018	−0.062	−0.092	0.076	0.025
**SLS-diff-EO/EC-R (s)**	−0.080	−0.176	−0.006	−0.125	−0.151	−0.133	−0.102	0.067	0.045
**SLS-diff-EO/EC-L (s)**	0.088	0.018	0.061	0.049	−0.010	−0.025	−0.074	−0.023	−0.007
**CoP-AP-R-EO (mm)**	0.076	0.104	0.006	0.181	0.140	0.113	0.163	−0.002	−0.043
**CoP-AP-L-EO (mm)**	0.132	0.272	0.031	0.280	0.251	0.133	0.151	0.067	0.038
**CoP-AP-R-EC (mm)**	0.115	0.019	0.198	−0.027	−0.033	0.159	0.208	0.014	0.034
**CoP-AP-L-EC (mm)**	0.080	0.060	0.059	0.071	0.044	−0.018	0.001	−0.053	−0.064
**CoP-ML-R-EO (mm)**	0.148	0.129	0.081	0.179	0.107	0.188	0.245	0.033	−0.107
**CoP-ML-L-EO (mm)**	0.060	0.085	0.027	0.090	0.075	0.053	0.058	−0.068	−0.174
**CoP-ML-R-EC (mm)**	0.100	0.118	0.089	0.137	0.111	−0.029	0.043	0.121	0.091
**CoP-ML-L-EC (mm)**	0.057	0.109	0.022	0.185	0.186	−0.147	−0.096	0.104	0.012
**CoP-MV-R-EO (mm/s)**	0.430 **	0.412 **	0.403 **	0.356 *	0.392 **	0.190	0.148	0.401 **	0.336 *
**CoP-MV-L-EO (mm/s)**	0.426 **	0.485 **	0.350 *	0.451 **	0.500 **	0.172	0.127	0.378 *	0.275
**CoP-MV-R-EC (mm/s)**	0.323 *	0.262	0.325 *	0.183	0.185	0.092	0.075	0.404 **	0.444 **
**CoP-MV-L-EC (mm/s)**	0.356 *	0.425 **	0.297 *	0.307 *	0.317 *	0.075	0.035	0.420 **	0.405 **

Note: Values are presented as Spearman’s correlation coefficients (ρ). TKJD-R—transverse knee joint diameter right; TKJD-L—transverse knee joint diameter left; TAJD-R—transverse ankle joint diameter right; TAJD-L—transverse ankle joint diameter left; SLS—Single-Leg Stance Test; R—right; L—left; EO—eyes open; EC—eyes closed; diff—difference; R/L—right/left; EO/EC—eyes open/eyes closed; s –seconds; mm—millimeters; mm/s—millimeters per second; CoP—center of pressure; AP—anteroposterior; ML—mediolateral; MV—mean velocity; BMI—body mass index; * *p* < 0.05; ** *p* < 0.01.

## Data Availability

The data presented in this study are available on request from the corresponding author.
